# STRATUM—structured framework for reporting, assessment, and translational utility of metagenomics

**DOI:** 10.1093/jaoacint/qsag085

**Published:** 2026-09-09

**Authors:** Joseph A Russell, Ishi Keenum, Karen Jarvis, Haley Sanderson, Michael D Sussman, Kamil Khanipov, Joe Lacirignola, Amy Xiao, Jonathan M Phillips, Malcolm Johns, Bala Ganesan, Cameron Parsons, Benjamin Davis, Shanmuga Sozhamannan

**Affiliations:** MRIGlobal, Gaithersburg, MD 20878, United States; Michigan Technological University, Civil, Environmental, & Geospatial Engineering, Houghton, MI 49931, United States; US Food and Drug Administration, Human Foods Program, College Park, MD 20740, United States; Agriculture and Agri-Food Canada, Biological Informatics Centre of Excellence, Information Systems Branch, Ottawa, ON K1A 0C6, Canada; U.S. Department of Agriculture, Livestock and Poultry Program, Standards and Specifications Division, Washington, DC 20250, United States; The University of Texas Medical Branch, Department of Pharmacology and Toxicology, The Microbiome Sequencing and Analysis Core, The Institute for Translational Sciences, Galveston, TX 77555, United States; MIT Lincoln Laboratory, Lexington, MA 02421, United States; MIT Lincoln Laboratory, Lexington, MA 02421, United States; Mayvin, Inc, Alexandria, VA 22312, United States; Mayvin, Inc, Alexandria, VA 22312, United States; Mars, Incorporated, McLean, VA 22101, United States; Merieux NutriSciences, Crete, IL 60417, United States; U.S. Environmental Protection Agency, Office of Research and Development, Cincinnati, OH 45268, United States; Joint Project Lead for Chemical, Biological, Radiological and Nuclear Enabling Technologies (JPL CBRN ET), Capability Program Executive for Chemical, Biological, Radiological and Nuclear Defense (CPE CBRND), Fort Detrick, MD 21702, United States; Joint Research and Development, Inc, Stafford, VA 22556, United States

## Abstract

**Background:**

Metagenomic next-generation sequencing (mNGS) enables broad, untargeted detection of pathogens and microbial signals across complex sample types. However, the diversity of operational contexts, from regulatory enforcement to exploratory discovery, challenges the defining of analytical or interpretive standards appropriate across all applications. Variability in laboratory practices, bioinformatic methods, and reporting conventions continues to limit consistency and decision-maker confidence in mNGS results.

**Objectives:**

We introduce Structured Framework for Reporting, Assessment, and Translational Utility of Metagenomics (STRATUM), a use-case–stratified framework that aligns QA, metadata reporting, and interpretive standards with the consequence and intended use of metagenomic sequencing outputs.

**Methods:**

STRATUM is organized around five representative biosurveillance use cases spanning public health, food safety, environmental monitoring, synthetic biology detection, and national security. A three-tier interpretive model calibrates analytical rigor, validation expectations, and reporting requirements to decision consequence; from high-consequence regulatory and clinical determinations (Tier 1), through operational surveillance (Tier 2), to exploratory and hypothesis-generating contexts (Tier 3).

**Results:**

The framework provides graduated guidance across key domains including sample preparation, sequencing design, controls and contamination governance, reference database curation, bioinformatics reproducibility, and multi-factor signal validation. Cross-cutting principles include explicit documentation of evidentiary bases, transparency in database and pipeline provenance, and defined escalation pathways when results transition between interpretive tiers.

**Conclusion:**

Realizing the operational potential of mNGS requires evidentiary standards responsive to decision context rather than fixed across applications. STRATUM offers a consequence-tiered model for quality and reporting in applied metagenomics, supporting reproducible, transparent, and defensible sequencing-based surveillance across public health and biodefense domains.

**Highlights:**

Introduction of STRATUM, a consequence-tiered evidentiary framework for applied metagenomics. Structured calibration of analytical rigor and reporting standards to decision context. Cross-use-case guidance spanning surveillance, regulatory, and national security applications.

Metagenomic next-generation sequencing (mNGS) has emerged as a transformative approach for the detection and characterization of biological signals across a wide range of biosurveillance applications, including wastewater monitoring, emergent outbreak investigation, environmental surveillance, detection of engineered biological constructs, and attribution of foodborne and zoonotic pathogens (1, 2). Unlike targeted molecular assays, mNGS enables broad, hypothesis-free interrogation of microbial communities, allowing simultaneous detection of known, novel, and unexpected organisms from complex samples (Figure 1). This breadth has positioned mNGSas a powerful complement to traditional PCR- and amplicon-based methods, including amplicon sequencing approaches for which minimal information standards have recently been established (3), particularly in settings where prior knowledge of targets is incomplete or evolving.

**Figure 1.**
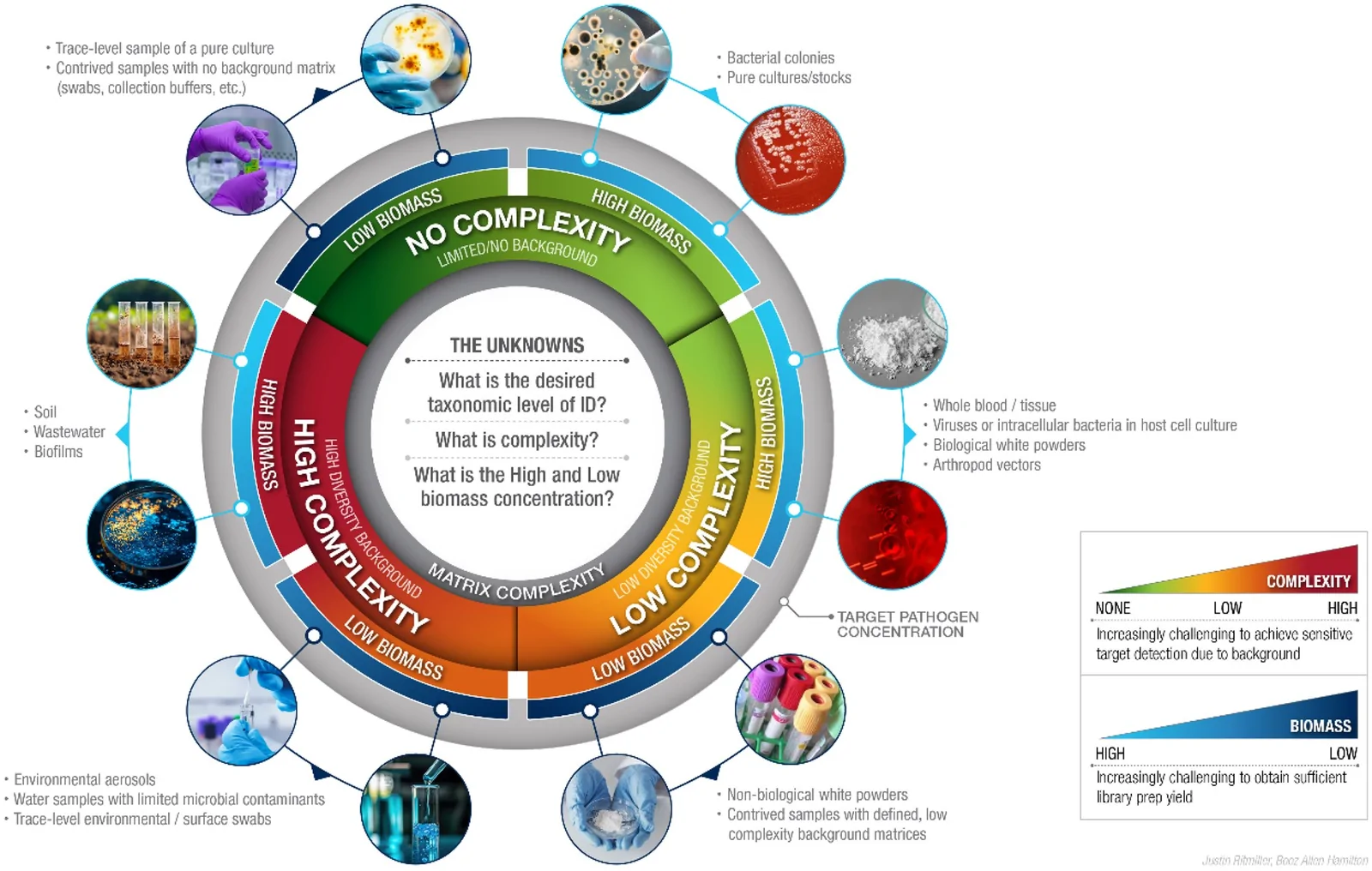
Conceptual classification of sample matrices by biological complexity and target biomass. Sample types are organized along two primary axes relevant to metagenomic sequencing performance and interpretation: matrix complexity (reflecting background biological diversity) and target biomass (reflecting abundance of the organism or genetic signal of interest). Representative sample types are shown for each region of the matrix. These categories are illustrative rather than prescriptive, as true background complexity and biomass can vary widely within a given sample type and are often poorly characterized a priori. The figure highlights how analytical difficulty increases as background complexity rises and target biomass decreases, and how required sequencing depth and data quality scale with the desired level of taxonomic or genomic resolution (e.g., genus/species detection versus strain- or SNP-level discrimination). This framework emphasizes that metagenomic workflow design and result interpretation must be tailored jointly to sample matrix, biomass, and intended use case.

However, the same attributes that make mNGS powerful also introduce substantial challenges for routine, operational use. Metagenomic workflows involve multi-step processes spanning sample collection, nucleic acid extraction, library preparation, sequencing, and computational analysis, each of which can introduce bias, variability, or contamination (4–6). In contrast to targeted approaches where LODs and performance characteristics are relatively well defined, mNGS outputs are highly dependent on sample matrix, sequencing depth, reference database composition, and bioinformatic methods (7–9). As a result, interpretation of mNGS data, particularly in high-background or low-biomass samples, remains inconsistent across laboratories and use cases. Without clear guidance on what constitutes sufficient evidence to support an actionable determination, there is a persistent risk of both false assurance and over-interpretation (10, 11).

Existing reporting standards and guidance documents have significantly advanced transparency and reproducibility in metagenomics research. Foundational metadata specifications such as MIGS (The minimum information about a genome sequence), MIMARKS (Minimum Information about a Marker Gene Sequence), and MIxS (Minimum Information about Any Sequence) established provenance and completeness requirements for genomic and metagenomic data (12, 13), while reporting checklists such as STREAMS (Standards for technical reporting in environmental and host-associated microbiome studies) and STORMS (The Strengthening the Organization and Reporting of Microbiome Studies) formalized disclosure expectations for microbiome studies (14, 15). In operational biosurveillance settings, decision-makers must evaluate not only whether a biological signal is present, but whether it is meaningful, credible, and warrants action, often under time constraints and with imperfect data. Critically, the level of analytical evidence required to support such a determination varies fundamentally with the consequences of the decision it informs: a regulatory recall demands a different evidentiary standard than a trend alert in a wastewater surveillance program, which in turn differs from an exploratory finding in a novel pathogen discovery effort. This contextual variability has been a persistent barrier to standardization. A detection event that demands immediate response in one setting may be inconsequential in another; a read-count threshold appropriate for high-biomass clinical specimens may be meaningless in a low-input environmental swab. The field has lacked a structured approach for calibrating analytical expectations, validation requirements, and interpretive posture to the operational context in which mNGS results will be used.

The objective of this document is to address this gap. Developed under the auspices of the AOAC INTERNATIONAL Stakeholder Panel on Agent Detection Assays (SPADA), the STRATUM framework provides guidance on data generation, quality assessment, metadata reporting, and interpretation of metagenomic results in support of confident decision-making. Rather than prescribing rigid methodological requirements or universal thresholds, the framework adopts a platform- and pipeline-agnostic approach organized around a three-tier interpretive structure that aligns analytical rigor with decision consequence: high-consequence regulatory and clinical applications (Tier 1), operational surveillance and early warning (Tier 2), and exploratory discovery (Tier 3). This tiered architecture reflects the principle that the standard of evidence required for a metagenomic finding should be proportional to the stakes of the action it may trigger.

To ground these principles in real-world practice, the framework was informed by five representative biosurveillance use cases:

- *Scenario 1*.—Wastewater-based monitoring of pathogen community trends;
- *Scenario 2*.—Strain-resolved tracking of emergent pathogens for outbreak response;
- *Scenario 3*.—Detection of synthetic or engineered biological constructs;
- *Scenario 4*.—Early detection of zoonotic spillover events in wildlife or sentinel populations;
- *Scenario 5*.—SNP-level attribution of foodborne pathogens for source tracing.

These use cases serve as illustrative examples through which shared analytical challenges (e.g., background discrimination, resolution requirements, confidence scoring, and thresholds for action) can be examined and generalized (Table 1). Further contextual descriptions of the applicability of each scenario can be found in Supplementary Scenario 1–Scenario 5.

**Table 1.** Summary of five cross-cutting metagenomic use case scenarios defined by our working group, where each scenario represents a distinct operational context for metagenomic methods, characterized by specific challenges, use case goals, and interpretive considerations

Scenario	Representative use- case description	Primary challenge	Key interpretation consideration
No. 1 Taxonomic surveillance of municipal wastewater (Supplementary Scenario 1)	Community pathogen monitoring via agnostic detection in wastewater to enable early warning and variant tracking.	High background complexity and low-abundance signals	Detection thresholds must balance sensitivity with signal interpretability across heterogeneous, time-varying matrices
No. 2 Functional gene detection in surface and air samples (Supplementary Scenario 2)	Detection of resistance, toxin, or engineered genes in low-biomass environmental samples for localized hazard monitoring.	Sparse DNA input and fragmented signals	Interpretation depends on contextual metadata and confidence in gene presence without organism-level attribution
No. 3 Synthetic biology detection in high-biomass, low-complexity samples (Supplementary Scenario 3)	Surveillance for engineered genetic features in high-biomass monocultures using reference-based and anomaly-driven metagenomics.	Novelty of sequences and evolving reference databases	Must incorporate anomaly detection, synthetic construct databases, and contextual review of unexpected features
No. 4 Arthropod-borne virus surveillance in vector populations (Supplementary Scenario 4)	Agnostic detection of known and novel viruses in field-collected arthropods enables early warning and lineage tracking for arboviruses before human cases appear.	Mixed host and vector nucleic acids; low pathogen abundance	Interpretation requires thresholds for genome coverage, lineage confidence, and temporal/geographic correlation
No. 5 Pathogen characterization for food safety and surveillance (Supplementary Scenario 5)	Metagenomic subtyping and linkage of pathogens within diverse food products. Supports regulatory traceback and recall decisions.	Complex matrices with potential background flora or contaminants	Requires high taxonomic resolution and strain-level confidence, with emphasis on reproducibility and source linkage

By articulating common interpretive and quality considerations across these diverse applications, this work aims to improve consistency, transparency, and trust in metagenomic sequencing outputs. The framework is intended to support laboratories, analysts, and decision-makers in understanding not only *what* was detected, but *how much confidence* can be placed in those detections and *how the results should be used* in operational contexts. Ultimately, we seek to facilitate responsible, reproducible, and actionable use of mNGS as it continues to transition from an emerging technology to a routine component of biosurveillance systems.

## Defining Actionable Signals in Metagenomics: Contextual Adaptation over Fixed Cutoffs

A persistent challenge in standardizing metagenomic methods is the contextual variability of what constitutes an *actionable signal*. Unlike conventional diagnostics, which rely on clear, quantitative thresholds (e.g., cycle threshold (Ct) values in PCR, colony forming units (CFU) counts in culture) to guide decisions, metagenomic sequencing produces probabilistic, multi-dimensional data that defy universal cutoffs. This variability stems from both the breadth of metagenomic applications and the differing stakes, timelines, and regulatory implications associated with each. As such, our framework does not advocate for a universal threshold or single-decision rule. Instead, it introduces a tiered interpretability framework (Table 2) that aligns analytical expectations with the consequences of decision-making in each scenario. No universal LOD defines Tier 1 adequacy; LOD expectations are use case and matrix dependent and must be characterized and reported relative to the specific analytical context rather than against a fixed numeric standard. High-consequence applications, such as outbreak confirmation, regulatory enforcement, or clinical treatment decisions require greater analytical rigor and stronger evidentiary standards for identification, quantification, and traceability. Lower-consequence applications such as passive surveillance, ecological studies, or anomaly detection in low-risk settings may tolerate more flexible approaches, provided that confidence levels and limitations are clearly communicated. By encouraging alignment between analytical burden and decision context, our framework enables flexibility without sacrificing trustworthiness. In doing so, we aim to harmonize expectations across diverse use cases while supporting the practical application of metagenomic sequencing in real-world biosurveillance systems.

**Table 2.** Tiered framework for interpreting metagenomic results based on use context and consequence^a^

Tier	Application context	Example scenarios	Decision consequence	Performance expectations	Recommended interpretation practices
Tier 1: High-consequence action	Regulatory, clinical, or national security decisions requiring high confidence	3 and 5	Actionable outcomes (e.g., recalls, treatment, lockdowns)	Highest analytical rigor: high specificity, sensitivity, LOD characterization, quantitative validation	Must include species/subspecies-level identification, documented limit of detection, orthogonal method confirmation
Tier 2: Operational surveillance	Early warning, exposure monitoring, facility QA/QC	1, 2, and 3	Informing alerts, triggers, or monitoring thresholds	Moderate rigor: consistent detection, repeatability, some quantitative metrics desirable	Use genus-level or functional gene presence; combine with trend analysis, historical baselines, or spike-in controls
Tier 3: Exploratory/discovery	Research, unknown threat detection, method development	1, 2, and 4	Low consequence, hypothesis-generating	Flexible: signal presence with supporting metadata	Report uncertainty; emphasize novelty or deviation from baseline rather than definitive ID; use database versioning annotations

a This framework provides a structured approach to guide performance expectations and interpretation practices aligned with the intended use of a metagenomic assay. As the stakes of decision-making increase, so too do the requirements for analytical rigor, specificity, validation, and confirmatory evidence. The table supports contextual flexibility while offering clear distinctions for actionability thresholds across regulatory, operational, and exploratory domains.

### Tier 1: High-consequence and Regulatory Applications

Tier 1 applications encompass metagenomic use cases in which analytical outcomes may directly inform clinical care, regulatory action, or public health and national security decision-making. In these contexts, both false-positive and false-negative results carry asymmetric and potentially irreversible consequences. Accordingly, Tier-1 applied metagenomics must be treated as a rigorously governed measurement system embedded within an evidentiary framework, rather than as a standalone discovery or screening tool (16–18). Across clinical diagnostics, food safety, and regulatory surveillance, the literature consistently demonstrates that interpretability and actionability in high-consequence settings are determined as much by pre-analytical design, contamination governance, and interpretive policy as by sequencing technology itself (10, 16). As a result, Tier 1 guidance emphasizes method validation, transparency, and conservative interpretation over maximal breadth or sensitivity.

Five interdependent governance domains define Tier 1 rigor, each detailed with domain-specific evidence review and operational recommendations in Supplementary T1. First, metagenomic workflows must be validated across *representative* matrix classes, because extraction target, host depletion strategy, and enrichment approach jointly determine sensitivity and specificity in ways that do not transfer across specimen types. Validation of a protocol against one food matrix or specimen class does not establish performance in another with meaningfully different composition, biomass, or inhibitor profile (10, 16, 17, 19). Second, sequencing platform and depth must be selected to support defensible inference with depth justified relative to intended LODs and evidentiary requirements (16, 20). Third, contamination governance must operate as a continuous surveillance function: end-to-end controls, longitudinal tracking of laboratory background signatures, and structured suppression or qualification rules are required to contextualize low-abundance signals in multiplexed and low-biomass workflows (11, 16, 21). Background contamination signatures are expected to vary between laboratories as a function of instrument use history, reagent sources, and physical environment; reproducibility derives from transparent monitoring and longitudinal characterization of laboratory-specific baselines (21), not from the assumption of uniform background across sites. Fourth, reference databases must be curated, version-controlled, and auditable, with documented inclusion criteria and known limitations disclosed as part of routine reporting, because persistent contamination and incorrect annotation in public repositories can propagate false signals into high-consequence analyses (22–26). Fifth, signal validation must be multifactorial: no universal read-count threshold defines actionability, and valid interpretation requires convergent evidence spanning genome coverage, locus consistency, control comparison, biological plausibility, and, where feasible, orthogonal confirmation (10, 16, 20).Confidence should emerge from the convergence of multiple, partially independent evidence domains rather than satisfaction of any single analytical metric. Accordingly, STRATUM treats interpretation as a graded evidentiary process rather than a binary determination, with reporting categories that distinguish confirmed findings, presumptive findings requiring additional confirmation, and detections that are not yet actionable (Table 3, Supplementary T1). Within STRATUM, a “confirmed” finding refers to a determination that satisfies the evidentiary standard required for the intended decision context through convergent evidence rather than any single analytical result. mNGS is capable of generating highly compelling analytical evidence. Whether that evidence constitutes a confirmed determination depends on the evidentiary standard required for the intended decision.

**Table 3.** Consequence-tiered documentation and governance framework for applied metagenomic sequencing^a^

Domain	Tier 1: High-consequence decision contexts	Tier 2: Operational surveillance and trend analysis	Tier 3: Exploratory and discovery contexts
Intended use and claim scope	Explicit statement of decision pathway supported (regulatory, clinical, enforcement). Analytical claims bounded to validated performance characteristics.	Statement of surveillance objective (trend detection, prioritization, early warning). Claims limited to comparative or longitudinal inference.	Statement of exploratory objective. Claims framed as hypothesis-generating.
Sample processing and extraction	Representative matrix-specific workflow defined and validated. Extraction target (DNA/RNA, whole-cell/cell-free) specified. Sensitivity, specificity, and dominant failure modes characterized.	Matrix-appropriate standardized workflow optimized for reproducibility across time points. Protocol stability maintained within surveillance intervals.	Workflow described sufficiently for interpretability and reproducibility. Performance characterization may be limited or evolving.
Sequencing strategy	Platform, read configuration, QC thresholds, and minimum usable reads specified. Depth justified relative to intended limit of detection and evidentiary requirements.	Platform and depth selected to ensure longitudinal consistency and adequate trend resolution. Emphasis on comparability over maximal sensitivity.	Platform and depth described. Breadth prioritized over validated detection thresholds.
Controls and contamination governance	End-to-end control framework (field blanks, extraction blanks, library blanks, spike-ins). Defined suppression or qualification rules when organisms appear in controls. Longitudinal tracking of laboratory background signatures.	Negative controls and internal standards used to characterize and normalize background signal. Control findings inform uncertainty and trend interpretation rather than automatic suppression.	Controls included where feasible; contamination risks acknowledged and documented.
Reference database governance	Database source, version, curation status, inclusion criteria, and known gaps disclosed. Version-controlled and auditable. Strain-level claims supported by alignment, coverage distribution, or phylogenetic placement.	Broad reference sets used for agnostic detection; version and source documented. Emphasis on consistency across surveillance intervals.	Database source disclosed. Assignments interpreted cautiously, especially for low-abundance or novel taxa.
Bioinformatic pipeline governance	Version-controlled, containerized or reproducible pipeline. Parameter stability enforced; changes require re-validation. Generation of reviewable evidence package (QC metrics, coverage plots, alignment summaries).	Stable, documented pipeline optimized for scalability and consistency. Major changes documented and benchmarked against prior baseline.	Pipeline described sufficiently for transparency. Methods may evolve iteratively.
Signal validation framework	Multi-factor evidence rubric: genome breadth and depth, locus consistency, control comparison, cross-sample contamination assessment, confirmatory testing triggers. No single read-count threshold treated as sufficient.	Signals interpreted relative to historical baselines and comparative trends. Validation emphasizes reproducibility across time or replicates.	Signals treated as provisional observations. Follow-up testing recommended before decision-making use.
Interpretation and reporting	Findings categorized (confirmed/presumptive pending confirmation/detected but not actionable). Explicit documentation of uncertainty sources (LOD, database gaps, matrix effects). Follow-up actions specified.	Results reported with uncertainty qualifiers and comparative framing. Emphasis on trend direction and magnitude rather than definitive attribution.	Findings clearly labeled as exploratory. Limitations and uncertainty prominently disclosed.
Escalation pathways	Not applicable (highest evidentiary tier).	Defined criteria for escalation to Tier- 1 confirmatory testing when thresholds of concern are met.	Defined criteria for structured follow-up under Tier 2 or Tier 1 frameworks.

a This table operationalizes STRATUM by outlining tier-specific documentation and interpretive requirements that support transparent, reproducible, and defensible use of metagenomics. While not prescribing fixed analytical thresholds, it defines structured minimum documentation principles aligned with the evidentiary demands of each decision context.

### Tier 2: Intermediate Risk/Operational Surveillance

Tier 2 applications encompass metagenomic use cases intended to support population-level situational awareness, trend detection, and prioritization across public health, environmental monitoring, and biodefense contexts, without directly triggering individual-level clinical decisions or regulatory enforcement. Representative examples include wastewater-based surveillance, environmental antimicrobial resistance monitoring, pathogen-agnostic outbreak tracking, and early warning activities aligned with Scenario 3. In contrast to Tier 1 applications, Tier 2 metagenomics prioritizes comparability, scalability, and longitudinal consistency over per-sample adjudication or maximal analytical sensitivity, while still requiring sufficient rigor to support actionable interpretation (27).

This shift in interpretive posture has practical consequences across every stage of the workflow, detailed with operational recommendations in Supplementary T2. Tie -2 workflows favor standardized, matrix-appropriate protocols that sacrifice absolute recovery efficiency in favor of reproducibility across sites and time points, because analytical outputs are interpreted primarily through relative change and trend analysis rather than binary detection (27, 28). Sequencing depth is selected to support relative abundance estimation and temporal dynamics, with platform and protocol consistency prioritized to preserve longitudinal interpretability; increasing depth beyond this point yields diminishing returns and does not compensate for upstream variability (9, 27). Control and benchmarking strategies support system-level normalization rather than exhaustive per-sample contamination adjudication, with taxa in controls interpreted through background subtraction and replication rather than strict suppression rules (9, 28). Reference databases are fixed within defined surveillance windows to prevent annotation drift from introducing artificial trends, and bioinformatics pipelines emphasize automation, containerization, and fixed parameters over analyst-driven optimization (9, 27, 28). Signal validation is inherently comparative: rather than universal read-count thresholds, Tier 2 interpretation emphasizes enrichment above background, concordance across replicates, temporal coherence, and alignment with orthogonal epidemiological or environmental indicators (27). Recent work relevant to Scenario 3 further illustrates this role. Studies on genetic engineering attribution demonstrate that engineered sequences may carry detectable, non-random design signatures that can support probabilistic inference and prioritization when analyzed at scale using curated reference corpora and calibrated models (29). However, these approaches are explicitly evidentiary and uncertainty-aware, reinforcing that Tier 2 metagenomics can contribute to hypothesis generation and situational awareness regarding potential synthetic origin, but does not, on its own, support deterministic attribution or enforcement conclusions. Accordingly, such methods align naturally with Tier 2 objectives when outputs are framed conservatively and integrated with broader investigative and intelligence workflows.

Operational surveillance systems must also anticipate how a novel detection will transition to higher-consequence interpretation. Airport-based metagenomic monitoring, now being deployed through the CDC’s Traveler-Based Genomic Surveillance program (30, 31), exemplifies a Tier 2 system designed with explicit escalation pathways to Tier 1 regulatory and clinical action.

### Tier 3: Exploratory/Discovery or Low-consequence Surveillance

Tier 3 applications encompass metagenomic use cases oriented toward hypothesis generation, method development, novel pathogen discovery, and ecological characterization, where outputs are not expected to directly inform clinical, regulatory, or operational decisions. Representative examples include academic microbiome surveys, novel virus discovery in wildlife reservoirs, pilot surveillance programs, and technology benchmarking studies. In these contexts, the primary value of metagenomics lies in its capacity to reveal unexpected biological signals, characterize community structure, and generate leads for further investigation rather than to support defensible, high-confidence determinations (8, 32).

Because the consequences of individual detection events are low and the analytical posture is discovery-oriented, Tier 3 guidance emphasizes documentation and transparency over prescriptive control. The objective is not to govern workflows to the degree required for Tier 1 or Tier 2 applications, but to ensure that methods, parameters, and interpretive context are recorded with sufficient fidelity to support reproducibility, reanalysis, and—critically—escalation to a higher tier if findings warrant further investigation. This documentation-first principle aligns with established metadata standards such as MIGS/MIMS and MIMARKS, which were developed explicitly to ensure that genomic and metagenomic data remain interpretable and reusable beyond their original analytical context (12–13).

This principle carries practical implications across the same governance domains defined for higher tiers, albeit with deliberately relaxed requirements. Workflows permit broad flexibility in sample preparation, platform selection, and analytical approach—ad hoc or novel protocols are acceptable, and platform choice is appropriately driven by cost, availability, and discovery objectives rather than regulatory defensibility (4, 7). Negative controls remain important, as contamination does not discriminate by application tier and can dominate signal in low-biomass and novel sample types (5, 6, 33); however, control results are used to contextualize findings rather than to enforce suppression rules. Reference databases should be broad and inclusive to maximize discovery potential, even where highly curated sets would be required for Tier 1 reporting (8), and bioinformatics pipelines may be iteratively optimized as part of method development, consistent with Findable, Accessible, Interoperable, and Reusable (FAIR) data principles (34). Across all domains, the unifying requirement is disclosure: what was done, with what tools and parameters, and with what known limitations.

Tier 3 signal validation does not require orthogonal confirmation or formal adjudication. The standard of evidence is plausibility and novelty rather than definitive identification, and results should be framed as hypothesis-generating with clear statements of analytical limitations including database scope, sequencing depth, and known biases (14). However, where a finding is sufficiently concerning or novel to warrant escalation—for example, detection of a potential select agent or an engineered construct—it should be flagged for confirmation under Tier 1 or Tier 2 standards before informing operational decisions. This escalation pathway is a deliberate design feature of the tiered framework: Tier 3 documentation requirements are set not only by the immediate analytical context but by the possibility that findings may later require higher-confidence adjudication. The value of a Tier 3 detection may not be immediately apparent; adequate documentation ensures that the path from discovery to action, should it become necessary, is not obstructed by gaps in provenance, methodology, or interpretive context.

### Tier-calibrated Governance

Across the five representative use cases and three evidentiary tiers, a consistent pattern emerges: applied metagenomics cannot be governed by a single universal checklist without either over-constraining low-consequence exploratory work or under-specifying high-consequence regulatory and clinical applications. This is not merely a temporary gap in validation science that additional research will eventually close; it is a structural characteristic of the field. The diversity of sample matrices, laboratory workflows, sequencing platforms, reference databases, and analytical pipelines makes universal numerical thresholds both scientifically unstable and operationally impractical. A read-count cutoff calibrated for one organism or sample matrix, for example, may systematically misclassify another, while validating every matrix-organism-workflow combination against a single global standard is infeasible. At the same time, insufficient documentation and inconsistent interpretive practices erode reproducibility, comparability, and decision-maker confidence. STRATUM’s central proposition is therefore not that evaluation criteria are unnecessary, but that the first step in addressing this tension should prioritize community agreement on evidentiary characteristics, structured documentation, and transparent interpretation rather than universal numerical thresholds. More generally, high-consequence metagenomic interpretation increasingly relies on the convergence of multiple independent evidence streams rather than any single analytical metric. Sequence abundance represents only one dimension of evidence and may be outweighed by orthogonal indicators such as genome coverage, genomic context, taxonomic consistency, control performance, replicate agreement, engineering signatures where applicable, or corroborating laboratory evidence. Confidence therefore emerges from the joint interpretation of these partially independent lines of evidence rather than satisfaction of a single numerical threshold. This principle is increasingly reflected in mature bioanalytical governance frameworks and in contemporary national metagenomic surveillance programs that emphasize standardized methods, metadata, and interpretive guidance over rigid analytical cutoffs (35, 36). STRATUM operationalizes this principle by separating analytical capability from evidentiary claim and calibrating documentation expectations, validation rigor, and reporting posture to the consequence level of the intended decision context. In Tier 1 settings, metagenomics is treated as a validated measurement system embedded within a defined evidentiary framework. In Tier 2 settings, emphasis shifts to longitudinal reproducibility, normalization, and bounded interpretive claims. Tier 3 settings prioritize transparency, disclosure, and hypothesis generation without overstating evidentiary strength.

Reference database governance illustrates this logic concretely. A recurring question is what qualifies a resource for use as a reference database across the metagenomics application tiers. Should eligibility be restricted to specific named databases, and how is that designation maintained as resources are updated, superseded, or newly created? STRATUM does not endorse specific databases, because no single public resource is positioned to serve as a universally authoritative reference across tiers, and the curation status, provenance, and disclosed limitations of available resources vary substantially and change over time (24, 26). A fixed list of approved databases would therefore either ossify quickly as the field evolves or implicitly endorse resources whose curation practices have not been evaluated against the claim being made. Instead, database suitability is governed by characteristics that scale with consequence: documented provenance and inclusion criteria, stable versioning, and disclosed curation status and limitations for Tier 1 use; consistency and version stability within defined surveillance windows for Tier 2 use; and disclosed scope sufficient to support later escalation for Tier 3 use. This same characteristics-based logic, rather than a fixed checklist of approved resources or universal thresholds, extends to the other governance domains summarized in Table 3: contamination control is governed by the presence of a defined control-and-monitoring framework rather than a specific suppression threshold; bioinformatic pipelines are governed by reproducibility and auditability rather than a mandated toolset; and signal validation is governed by convergent, multi-factor evidence rather than a single read-count cutoff. Confidence emerges from the combined weight of multiple evidence domains rather than any individual criterion.

Table 3 translates STRATUM’s tier-calibrated governance principles into practical documentation and interpretive expectations across the major domains of metagenomic analysis, including sample processing, sequencing strategy, contamination governance, reference database provenance, bioinformatic reproducibility, signal validation, and reporting posture. Together, these domains provide a structured, auditable framework for evaluating analytical confidence while allowing implementation to remain appropriate to the validated workflow and intended decision context. STRATUM governs evidentiary principles rather than specific technologies, and thus, it is inherently adaptable to future advances in sequencing platforms, bioinformatic methods, AI-assisted interpretation, and synthetic biology detection. As analytical capabilities evolve, the framework’s consequence-tiered evidentiary logic remains applicable without requiring fundamental restructuring, allowing innovation in methodology while preserving consistency in interpretation and governance.

## Conclusions

Applied metagenomics now spans clinical diagnostics, food safety enforcement, environmental surveillance, synthetic biology detection, and national security monitoring. Despite rapid technological maturation, interpretive governance has lagged behind analytical capability. Efforts to impose universal minimum-information criteria have struggled against the diversity of matrices, workflows, and decision contexts in which sequencing is deployed. It is our hope that future work will establish more granular specificity of validation requirements within application tiers and specific applied use cases, but this is outside of the scope of STRATUM’s contribution.

Rather, STRATUM addresses an “up-stream” organizational requirement providing a structured, consequence-tiered framework that aligns documentation, validation rigor, and interpretive posture with the stakes of the intended application. By distinguishing high-consequence evidentiary use from operational surveillance and exploratory discovery, it enables reproducible and transparent use of metagenomics without constraining innovation or overstating analytical certainty. As metagenomic sequencing becomes increasingly embedded in public health and biodefense infrastructures, defensible deployment will depend not solely on sequencing depth or classifier performance, but on calibrated governance. STRATUM offers a scalable architecture for that governance, supporting decision-relevant, transparent, and context-aware application of metagenomics across diverse domains.

## Supplementary Material

qsag085_Supplementary_Data

## Disclaimers

The views expressed in this paper are those of the authors and do not necessarily represent the views or official position of Defense Biological Product Assurance Office (DBPAO), Capability Program Executive (CPE), Department of War, U.S. Food and Drug Administration (FDA), U.S. Department of Agriculture (USDA), U.S. Department of Homeland Security (DHS), or U.S. Environmental Protection Agency (EPA). The authors K.J. (retired), M.S., B.D., and S.S. are US Government employees, and this work was prepared as part of their official duties. Title 17 U.S.C. §105 provides that “Copyright protection under this title is not available for any work of the United States Government.” Title 17 U.S.C. §101 defines a U.S. Government work as a work prepared by a military service member or employee of the U.S. Government as part of that person’s official duties. Certain commercial equipment, instruments, or materials are identified in this paper to foster understanding. Such identification does not imply recommendation or endorsement, nor does it imply that the materials or equipment identified are necessarily the best available for the purpose. The author H.S. is a Canadian Government employee. ^©^His Majesty the King in Right of Canada, as represented by the Minister of Agriculture and Agri-Food.
