# Supplementary material for: STRATUM—structured framework for reporting, assessment, and translational utility of metagenomics
Source: J AOAC Int. 2026 Sep 9;109(6):1042–51. doi: 10.1093/jaoacint/qsag085 (PMC13732714; doi:10.1093/jaoacint/qsag085)
Supplement: Supplementary file 1 [file qsag085_supplementary_data.docx]

**STRATUM SUPPLEMENTARY**

**SUPPLEMENTARY S1 —**

**Scenario 1: Taxonomic Surveillance of Municipal Wastewater**

**Why Metagenomics?**
Municipal wastewater aggregates biological material from large populations, making it a powerful matrix for community-level pathogen surveillance. While targeted assays such as qPCR are effective for tracking known organisms, they are inherently constrained to predefined targets. Metagenomic sequencing enables agnostic detection across bacteria, viruses, fungi, and parasites within a single assay, supporting discovery of emerging pathogens, characterization of antimicrobial resistance, and broader contextualization of microbial signals over time. This breadth makes metagenomics particularly attractive for surveillance applications where the question is not necessarily whether a specific known pathogen is present, but whether emerging/unknown biothreats may be circulating.

**Why It Matters**
Wastewater-based surveillance informs public health decision-making by providing early indicators of disease trends before clinical cases are detected. Public health agencies, environmental authorities, and national security organizations increasingly view wastewater metagenomics as a complementary layer within broader biosurveillance systems (Philo et al. 2023, Child et al. 2023, Grimm et al. 2026). Beyond outbreak monitoring, wastewater sequencing can inform treatment efficacy, detect shifts in microbial ecology, and identify anomalous signals that warrant further investigation.

**What Makes It Hard**
Wastewater is an extremely complex and heterogeneous matrix, with high background nucleic acid abundance that can obscure low-level pathogens of interest. Detection sensitivity is strongly influenced by sample type (e.g., influent versus solids), collection strategy, concentration and extraction methods, sequencing depth, and temporal variability. Unlike targeted assays, metagenomic limits of detection and decision thresholds are not intrinsic and must be empirically established. Contamination from reagents, laboratory environments, or reference databases poses persistent risks, as does biological variation driven by flow rate, seasonality, and population behavior. Interpreting whether observed changes represent meaningful public health signals or normal background fluctuation remains a central challenge.

**Platform and Analysis Considerations**
Short-read sequencing provides high-confidence taxonomic detection and remains the backbone of most wastewater metagenomic workflows, while long-read or hybrid approaches can add contextual resolution for antimicrobial resistance genes and genomic linkage when required. Analytical rigor depends on defined sequencing depth targets, robust negative and positive controls, curated reference databases, and reproducible bioinformatic pipelines. Normalization strategies and longitudinal baselines are critical for distinguishing true signal from noise and enabling meaningful temporal comparisons.

**So What?**
Wastewater metagenomics illustrates both the promise and complexity of applying mNGS for population-level surveillance. It highlights the need for standardized controls, explicit detection thresholds, transparent metadata, and clear interpretive frameworks to support actionable decisions. These requirements directly inform the cross-cutting recommendations presented in STRATUM, which aim to ensure that metagenomic results are both technically sound and operationally meaningful across diverse surveillance contexts.

**SUPPLEMENTARY S2 —**

**Scenario 2: Functional Gene Detection in Surface and Air Samples**

**Why Metagenomics?**
Surface and air samples are often low-biomass and inhibitor-rich, presenting unique challenges for biosurveillance. Traditional methods such as culture or PCR offer limited scope, focusing on known organisms or genes. Metagenomics enables broader, agnostic detection of functional markers such as antimicrobial resistance genes, virulence factors, or synthetic biology elements, even in the absence of viable organisms. This capability is critical in scenarios where rapid environmental assessment is needed to guide response.

**Why It Matters**
Metagenomic surveillance of environmental surfaces and air filters can reveal functional threats like resistance or toxin genes before infections emerge or contamination spreads. This is particularly relevant for early warning in biodefense, attribution in suspected bioterrorism events, or continuous monitoring in hospitals, transit hubs, or production facilities. Unlike passive wastewater monitoring, these matrices support proactive, localized hazard detection.

**What Makes It Hard**
The challenges are multifaceted: low DNA yields raise contamination risks, and environmental DNA may be degraded or fragmented. Human DNA contamination and temporal variation in gene abundance further complicate interpretation. False positives are a concern due to sparse signal, and distinguishing meaningful markers from ambient background requires careful database selection, stringent controls, and replicate validation.

**Platform and Analysis Considerations**
Short-read sequencing remains the most reliable option for ultra-low-input samples, while long reads can contextualize gene architecture if sufficient DNA is available. A hybrid approach may offer the best of both. Regardless of platform, robust analytics with spike-in controls, curated gene databases (e.g., Alcock et al. 2023, Dong et al. 2024, Zhou et al. 2025), and strict alignment thresholds are essential to ensure confidence and reproducibility in functional gene calls.

**So What?**
Similar to wastewater, environmental surface and air metagenomics allows detection of functional biodefense signals such as virulence or resistance genes before they manifest as health threats. With proper controls and analytic rigor, this approach can complement targeted methods to expand early warning capabilities, support attribution efforts, and offer decision-relevant insight into evolving biohazards.

**SUPPLEMENTARY S3 —**

**Scenario 3: Metagenomics-Based Synthetic Biology Detection in High-Biomass, Low-Complexity Samples**

**Why Metagenomics?**

Metagenomic sequencing is defined as the minimum standard for detecting synthetic biology in high-biomass, low-complexity samples such as monocultures, industrial fermenters, engineered probiotics, and biomanufacturing systems. Unlike targeted assays that depend on prior knowledge of specific genetic features, metagenomics captures the full nucleic acid content of a sample, enabling both reference-based detection of known synthetic elements (e.g., promoters, vector backbones, selectable markers) and agnostic identification of engineered anomalies. These anomalies may include codon usage bias, atypical GC content transitions, or chimeric junctions that span multiple taxa. In environments where biomass is abundant but taxonomic diversity is low, engineered features may be subtle, intentionally obscured, or absent from reference databases, making unbiased metagenomic interrogation uniquely suited for synthetic engineering detection.

**Why It Matters**

Accurate detection of synthetic genetic modifications is critical for national security, public health, regulatory oversight, and industrial quality assurance. Intelligence and defense organizations rely on early warning and attribution capabilities for engineered biothreats, while public health agencies must assess the safety and provenance of engineered microbes used in therapeutics or environmental applications. Regulatory bodies such as FDA and USDA require confidence in genetic composition for approval, monitoring, and enforcement, and industrial biomanufacturing facilities must ensure the integrity of production strains and inputs. Scenario #3 establishes a common analytical framework that supports interoperable, reproducible assessments of synthetic engineering across these mission-critical domains.

**What Makes It Hard**

Detecting synthetic biology features is complicated by modern engineering practices that can evade straightforward identification. Codon optimization can erase recognizable similarity to natural genes while preserving function, and advanced editing approaches enable scarless insertions that leave no obvious junctions. Increasingly, AI-designed genes and proteins may retain dangerous biological activity while diverging substantially from known sequences, undermining homology-based detection (Whittman et al. 2025). Subtle modifications such as single-nucleotide edits embedded within common laboratory strains further challenge attribution, and current origin-assignment tools remain limited by incomplete and inconsistently curated reference repositories (Wang et al. 2021). These challenges require standards that explicitly accommodate both current technical constraints and emerging design strategies.

**Platform and Analysis Considerations**

Scenario #3 defines complementary roles for sequencing platforms and analytical approaches. Short-read sequencing is established as the baseline requirement due to its low error rate and suitability for detecting small edits, codon-optimized genes, and subtle junction-spanning reads. Long-read sequencing is required when reconstruction of full-length inserts, plasmids, or large structural rearrangements is necessary, particularly in monoculture or biomanufacturing contexts. Hybrid approaches are preferred, combining nucleotide-level precision with structural resolution. Analytically, pipelines must integrate curated reference databases, rule-based detection of synthetic parts, statistical anomaly detection (e.g., codon usage or GC shifts), and functional risk classification using biosafety and biosecurity ontologies. Rigorous contamination controls, including blanks and spike-ins, are mandatory, and human adjudication through visualization and review of flagged features is required to validate computational outputs.

**So What**

Near-term standards require curated synthetic biology databases, explicit motif- and part-level detection, anomaly screening, comprehensive metadata, and contamination control. Forward-compatible standards mandate pipeline architectures that can incorporate AI-native anomaly detection, probabilistic scoring, and evaluation of stealth or scarless constructs. Benchmarking against standardized datasets containing engineered homologs of concern, realistic monoculture backgrounds, and diverse design paradigms is required to validate performance. Together, these requirements define unbiased metagenomic sequencing (supported by curated references, anomaly detection, functional risk classification, and human-in-the-loop review) as the minimum standard for synthetic engineering surveillance in high-biomass, low-complexity samples.

**SUPPLEMENTARY S4 —**

**Scenario 4: Arthropod-Borne Virus Surveillance in Vector Populations**

**Why Metagenomics for This Use Case**

Arthropod-borne viruses circulate within complex vector–host–environment systems, where emergence is often unpredictable and driven by ecological change, vector migration, or viral evolution. Metagenomic sequencing enables agnostic detection of known and novel arboviruses directly from mosquito and tick populations, supporting early warning, lineage tracking, and characterization of genetic features associated with transmissibility or virulence. Unlike targeted assays, metagenomics can detect unexpected viruses, divergent strains, and mixed infections without prior assumptions, making it particularly valuable for monitoring sylvatic transmission cycles and spillover risk.

**Why It Matters / Primary Stakeholders**

Primary stakeholders include public health agencies, vector control programs, biodefense and national security organizations, and academic researchers studying emerging infectious diseases. For public health and defense, vector-based metagenomics provides an upstream surveillance layer that can precede human or animal cases. For vector control programs, genomic prevalence data informs geographic prioritization and intervention strategies. For researchers, these data support genomic epidemiology, evolutionary analysis, and understanding of vector competence and pathogen ecology.

**What Makes This Hard**

Arboviruses are typically present at low abundance relative to vector nucleic acids, requiring highly sensitive extraction and sequencing workflows. Host/vector DNA and RNA can overwhelm viral signal, necessitating enrichment or depletion strategies that introduce bias if not carefully documented (e.g., Chan et al. 2022). Viral RNA degradation, fragmented genomes, and within-vector diversity complicate assembly and variant calling. Reference genome gaps for many arboviruses limit confident taxonomic resolution, particularly for novel or divergent strains. Interpretation is further complicated by uncertainty around viability, infectivity, and the relationship between viral detection in vectors and actual transmission risk.

**Platform and Analytical Considerations**

Short-read sequencing provides high-confidence detection and variant calling for fragmented viral genomes, while long-read sequencing offers contextual information such as genome structure, methylation, and linkage of mutations, albeit with higher input requirements and error rates. Hybrid strategies can mitigate these tradeoffs when sufficient material is available. Analytical workflows must incorporate host depletion, rigorous contamination controls, curated viral reference databases, and phylogenetic comparison against public datasets to contextualize findings. Variant interpretation should account for within-vector diversity and stochastic sampling effects, particularly in pooled specimens.

**How This Scenario Informs Broader Recommendations**

This use case highlights cross-cutting needs for transparent documentation of enrichment and depletion methods, clear analytical thresholds for detection and variant calling, and standardized metadata capture (e.g., vector species, collection method, geolocation, and time). It underscores the importance of separating detection capability from risk interpretation, and of clearly articulating what constitutes an actionable signal versus background viral diversity. These considerations directly inform STRATUM guidance on detection confidence, database curation, contamination control, and interpretation frameworks across surveillance contexts.

**SUPPLEMENTARY S5 —**

**Scenario 5: Pathogen Characterization for Food Safety & Surveillance**

**Why Metagenomics?**

Metagenomics offers a powerful supplement to traditional food safety workflows by enabling broad detection of pathogenic organisms and their clonal variants within complex food matrices. It provides additional resolution for source attribution through in silico serotyping, MLST, and SNP-based characterization (e.g., Zolfo et al. 2016). Unlike culture-based or PCR methods that require prior knowledge of the target, metagenomics can operate agnostically, supporting outbreak tracing even when the specific pathogen or subtype is not initially known.

**Why It Matters**

High-profile outbreaks involving *Salmonella spp.*, *Cronobacter spp.*, and *Listeria spp.* have highlighted the need for faster, more scalable pathogen characterization methods. Regulatory decision-making (e.g., issuing a recall) often hinges not only on identifying the presence of a pathogen, but on linking it to a known outbreak strain. Metagenomics can facilitate this, especially when integrated with enrichment strategies that enhance signal from low-abundance organisms. Quasi-metagenomic approaches (such as sequencing from enriched or resuscitated cultures) enable higher sensitivity while preserving taxonomic context.

**What Makes It Hard**

Food matrices are among the most challenging substrates for metagenomics due to their heterogeneity, high background microbial content, and presence of PCR inhibitors. There is no one-size-fits-all extraction or sequencing workflow across food types. Solid vs. liquid foods, for example, require different concentration and lysis techniques. Downstream analysis is complicated by incomplete or misannotated reference databases, and performance varies widely by pathogen (e.g., *Escherichia coli* may require custom reference databases for confident strain-level detection). Moreover, metagenomic readouts are proportional rather than quantitative, making actionable thresholds ambiguous without baseline matrix data.

**Platform and Analysis Considerations**

A typical workflow may involve sample enrichment (e.g., selective culture, live capture, or nucleic acid capture), followed by DNA extraction (with controls for extraction efficiency), library preparation, and high-throughput sequencing. Raw reads undergo quality filtering, optional assembly, and taxonomic classification using tools such as Kraken2, KMA, or Mash, among others; these are cited as representative examples of available approaches and are not intended as an exhaustive or endorsed list. However, the choice of reference database is critical. GenBank and NCBI nt may contain errors or incomplete coverage; curated databases like RefSeq or custom pathogen-specific references are preferred. In silico serotyping and MLST analysis can be performed post hoc for regulatory reporting and source tracking. Tools such as *BetterCallSal* (Konganti et al. 2023) exemplify this category of application; equivalent or successor tools performing the same analytical function are equally applicable where performance has been appropriately characterized. All bioinformatic tools referenced throughout this framework are cited as illustrative examples of available approaches. Their mention does not constitute endorsement, and equivalent tools performing equivalent analytical functions are applicable where performance characteristics have been appropriately established. AI/ML tools are emerging to help prioritize actionable calls, but database transparency and reproducibility remain core challenges.

**So What?**

Metagenomics is best positioned today as a funnel that prioritizes detailed analysis on a subset of samples identified through screening (e.g., ~300 samples narrowed to 20 positive hits). It is unlikely to replace targeted detection methods for routine surveillance but offers unmatched value in outbreak response, when strain-level attribution and detection of novel or VBNC (viable but non-culturable) organisms are essential. To become more broadly actionable, the field must establish baseline profiles for common food matrices, define standards for detection and quantification thresholds, and clarify how metagenomics informs regulatory decisions (e.g., when to initiate a recall). Until then, its greatest utility may be in triage, source tracking, and method development, especially when paired with culture enrichment or quasi-metagenomic pipelines that focus on specific pathogens.

**SUPPLEMENTARY T1**

**Tier-1 applied metagenomics: the standard of evidence**

Tier-1 applications place metagenomic results into decision pathways with asymmetric consequences. False positives can produce inappropriate regulatory action, misallocation of response resources, or unnecessary clinical intervention, while false negatives can delay containment or treatment. In this context, Tier-1 metagenomics should be treated as a validated measurement system embedded within an evidentiary framework, not as a generic “agnostic detection” capability (Chiu and Miller 2019; Wilson et al. 2019; Billington et al. 2022; Wang et al. 2025). Across both clinical and food-safety domains, performance is governed as much by pre-analytical design, contamination control, and interpretation policy as by sequencing technology itself (Chiu and Miller 2019; Graf et al. 2020; Sun et al. 2025). Tier-1 recommendations therefore emphasize method governance: what must be controlled, documented, validated, and disclosed to make conclusions defensible.

**Sample preparation and extraction: matrix-specific designs, not one-size-fits-all**

Tier-1 workflows must be validated per matrix and specimen type. Host/background nucleic acid burden, inhibitory substances, and organism biology determine whether whole-cell extraction, cell-free extraction, enrichment, host depletion, or targeted capture is appropriate, and no single strategy is defensible across all Tier-1 contexts (Chiu and Miller 2019; Wilson et al. 2019; Sun et al. 2025). Comparative clinical body-fluid analyses demonstrate that whole-cell versus cell-free approaches can invert sensitivity depending on specimen type, and that extreme host background can render an otherwise robust workflow ineffective (Sun et al. 2025). Food-safety literature similarly emphasizes that pathogen detection in complex matrices frequently requires quasi-metagenomic approaches such as enrichment or targeted separation, while acknowledging that these steps bias community composition and complicate downstream inference (Billington et al. 2022; Wang et al. 2025).

**Recommendation (Tier-1):**

Workflows must specify extraction target (whole-cell, cell-free, RNA, DNA), pre-treatment steps (lysis, depletion, enrichment), and input requirements, with validation across representative matrix classes that characterizes sensitivity, specificity, and dominant failure modes. Validation within a matrix class does not require enumeration of every substrate variant (e.g., every cheese variety, every food product formulation, every environmental substrate type, etc.) but should demonstrate performance across classes that differ meaningfully in background complexity, biomass, or inhibitor profile (Chiu and Miller 2019; Sun et al. 2025; Billington et al. 2022). The impossibility of exhaustive per-matrix validation is itself a founding observation of the STRATUM framework. This reason is precisely why consequence-tiered governance is necessary rather than a single universal standard. For Tier-1 food safety applications, matrix class boundaries should align with existing regulatory validation frameworks such as FDA Bacteriological Analytical Manual categories, USDA FSIS method validation guidance, or ISO 16140 food microbiology validation standards where applicable (e.g., Ritter et al. 2009), rather than requiring laboratories to construct new taxonomies. Validation within a recognized matrix category establishes the scope of defensible claims for that category; results from a workflow validated against one category should not be extended to another without documented bridging studies. This does not impose new validation burdens beyond what existing regulatory frameworks already require for conventional microbiological methods. Rather, it requires that those existing scope boundaries be explicitly stated as part of metagenomic reporting.

**Sequencing strategy: depth and accuracy are necessary but insufficient**

Sequencing platform selection in Tier-1 applications should be driven by the regulatory or clinical claim being made, rather than discovery breadth alone. Short-read platforms remain the most defensible default for high-consequence taxonomic detection because of their mature error profiles, stability, and regulatory familiarity (Chiu and Miller 2019; Wilson et al. 2019).

Although increased sequencing depth improves pathogen recovery, it does not resolve interpretive risk when background contamination, index bleed, or low-biomass artifacts are present. This limitation is evident in comparative studies where sensitivity gains are accompanied by reduced specificity, even under structured analytical thresholds (Graf et al. 2020; Sun et al. 2025). In respiratory virus surveillance, validated clinical deployments emphasize automation, turnaround time, and integrated computational pipelines as components of deployable systems, rather than platform novelty alone (Tan et al. 2024).

**Recommendation (Tier-1):** Specify platform, read configuration, minimum usable reads after quality control, and multiplexing limits. Depth targets should be justified relative to intended limits of detection and downstream evidentiary requirements (e.g., genome coverage, assembly, or genotyping), not throughput alone. No universal limit of detection exists across STRATUM use cases; acceptable LoD is determined by the intended application, the consequence of the decision it supports, and the matrix and organism class under investigation. A Tier-1 food safety workflow targeting strain-level attribution operates under different LoD expectations than a Tier-1 clinical metagenomics application, and neither can be governed by a single numeric standard. LoD characterization should therefore be performed and reported relative to the representative matrix class and representative organism class for which the workflow is validated, with reference to published performance benchmarks where available rather than universal cutoff values (Chiu and Miller 2019; Tan et al. 2024; Sun et al. 2025).

**Controls and contamination governance: continuous monitoring is a Tier-1 requirement**

Tier-1 metagenomics must treat low-level contamination and index-related artifacts as persistent hazards rather than anomalies. Clinical and food-safety literature converge on the need for end-to-end controls spanning sampling blanks, extraction blanks, library blanks, and sequencing controls, coupled to explicit decision rules governing reportability when organisms appear in negative controls (Chiu and Miller 2019; Graf et al. 2020; Billington et al. 2022; Wang et al. 2025). Operational respiratory virus surveillance further demonstrates that high predictive agreement with orthogonal diagnostics depends on discrepancy testing, adjudication, and stable background baselines, all of which implicitly rely on robust control architectures (Tan et al. 2024).

**Recommendation (Tier-1):** Implement a control framework that (i) detects contamination sources, (ii) quantifies run-specific background, and (iii) enforces suppression or qualification rules when taxa are present in controls. Controls should be tracked longitudinally to establish laboratory-specific contaminant signatures and batch effects. Laboratory-specific background signatures are an expected feature of operational sequencing environments rather than a framework failure. Instruments used across multiple applications, reagent lots, and physical spaces will accumulate distinct contamination profiles that differ between sites. Reproducibility in Tier-1 metagenomics therefore does not require identical background signatures across laboratories; it requires that each laboratory's background be characterized, monitored, and transparently reported so that low-abundance signals can be contextualized against the appropriate baseline. Inter-laboratory comparability is achieved through transparent documentation and structured control practices, not through the elimination of laboratory-specific variation (Chiu and Miller 2019; Graf et al. 2020; Billington et al. 2022; Wang et al. 2025, Price and Russell 2023, Li et al. 2020).

**Reference databases: provenance, versioning, and curation must be explicit**

In Tier-1 metagenomic applications, reference databases function as analytical infrastructure rather than background resources. Because taxonomic assignments, abundance estimates, and downstream interpretations are necessarily conditioned on reference content, database composition and curation status directly influence both sensitivity and specificity in high-consequence settings. The literature now clearly establishes that publicly available sequence repositories contain nontrivial levels of contamination, mis-annotation, and taxonomic inconsistency that can propagate false signals into applied metagenomic analyses if left unmanaged (Field et al. 2008; Lu and Salzberg 2018; Lupo et al. 2021). Early standards efforts such as the Minimum Information about a Genome Sequence (MIGS) specification emphasized metadata completeness, provenance, and transparency as prerequisites for meaningful reuse of genomic data, explicitly recognizing that interpretability depends on context as much as sequence content (Field et al. 2008). Subsequent extensions to metagenome-assembled and single-cell genomes reinforced this principle, introducing explicit quality and completeness criteria while acknowledging persistent uncertainty in reference datasets derived from heterogeneous sources. However, these standards were developed primarily to support data sharing and comparative genomics, rather than regulatory or clinical decision-making.

More recent analyses demonstrate that contamination in reference databases is both pervasive and method-dependent. Systematic evaluations of RefSeq and related repositories show that even low aggregate contamination rates can yield high downstream impact, as a single misannotated or chimeric genome may generate spurious taxonomic assignments across many samples (Breitwieser et al. 2019; Lupo et al. 2021). Importantly, different contamination-detection methods frequently produce discordant results, reflecting algorithmic assumptions and the recursive dependence of detection tools on imperfect reference sets. These findings undermine the assumption that any single public database, or single contamination-screening approach, can be treated as authoritative for Tier-1 reporting.

Reference databases used in Tier-1 applications must meet explicit governance criteria rather than belonging to any approved list. The field currently lacks a universally authoritative reference corpus. Public sequence repositories function primarily as submitter-driven data archives rather than curated reference resources, and the provenance, accuracy, and curation status of individual records varies substantially and is not independently validated at the point of submission (Jacobs 2026; Lu and Salzberg 2018; Lupo et al. 2021). Accordingly, Tier-1 database suitability should be evaluated against the following characteristics: documented inclusion criteria and exclusion rationale; explicit versioning with stable, citable build identifiers; traceable data provenance linking sequences to authenticated physical source materials where applicable; defined contamination-screening procedures and known limitations disclosed; and governance structures that support ongoing curation rather than static deposition (Wright et al. 2024; Nguyen et al. 2024; Jacobs 2026; Harmon et al. 2023). Databases meeting these criteria (e.g, institutional collections such as the ATCC Genome Portal, regulatory-grade resources such as FDA-ARGOS, or curated national type-strain repositories) may be appropriate for Tier-1 use when their scope, curation status, and known gaps are explicitly disclosed as part of reporting. The question for Tier-1 governance is not which databases are approved, but whether a database's provenance and curation practices are sufficient to support the evidentiary claims being made.

**Recommendation (Tier-1):** Tier-1 reports must therefore document: 1) the specific database name, build version, and date of access; 2) the curation and contamination-screening methods applied to that build; 3) known taxonomic gaps or limitations relevant to the assay's target organisms; and 4) any supplemental databases used to extend coverage. Strain-level or functional claims must be supported by evidence beyond initial k-mer or alignment-based classification — including alignment identity distributions, breadth of genome coverage, and, where available, phylogenetic placement against type strain references.

**Bioinformatics pipelines: auditability and interpretability are part of analytical validity**

Tier-1 pipelines must be stable, reviewable, and auditable. The objective is not rapid classification alone, but defensible inference. Clinical metagenomics guidance emphasizes expert review of interpretable artifacts (e.g., coverage plots, alignments, and phylogenetic context) as essential for high-consequence reporting (Wilson et al. 2019). Validated respiratory virus workflows further demonstrate that pipeline design, including host subtraction, assembly, and translated-search components, is integral to assay capability, particularly for novel or divergent pathogens (Tan et al. 2024). In food-safety contexts, the absence of standardized, widely accepted metagenomic pipelines is repeatedly identified as a barrier to regulatory integration (Billington et al. 2022; Wang et al. 2025).

**Recommendation (Tier-1):** Pipelines must be version-controlled, containerized or equivalently reproducible, and capable of generating reviewable evidence packages (QC metrics, host-read subtraction summaries, confidence-qualified taxonomic assignments, and alignment/coverage visualizations). Parameter changes require controlled re-validation prior to Tier-1 use (Chiu and Miller 2019; Tan et al. 2024; Billington et al. 2022).

**Signal validation: multi-factor evidence rules, not single thresholds**

Tier-1 validation should treat metagenomic output as probabilistic evidence requiring corroboration. Studies employing explicit read-count or z-score thresholds demonstrate the utility of transparent decision rules, but also show that specificity can remain limited even with high depth and structured criteria (Sun et al. 2025). Clinical metagenomics reviews emphasize that no universal read threshold exists; validity depends on genome breadth, distribution across loci, control comparison, and biological plausibility, with orthogonal testing recommended for unexpected or high-stakes findings (Chiu and Miller 2019; Wilson et al. 2019).

In respiratory virus detection, comparative studies indicate that high concordance with established assays is achieved when manual confirmation and adjudication are incorporated, supporting an evidence-based rather than threshold-only model (Graf et al. 2020; Tan et al. 2024).

**Recommendation (Tier-1):** Define a signal rubric incorporating (i) genome-level breadth and depth, (ii) locus consistency, (iii) control and background comparison, (iv) cross-sample contamination checks, and (v) confirmatory testing triggers. Read-count cutoffs may be included but should not be treated as sufficient conditions for action (Chiu and Miller 2019; Sun et al. 2025; Tan et al. 2024).

**Interpretation and reporting: regulatory-grade communication of uncertainty**

Tier-1 reporting must be designed for downstream decision-makers. Food-safety reviews emphasize that metagenomics does not map cleanly onto binary regulatory frameworks and advocate for risk-based interpretation that distinguishes screening signals from confirmed findings (Billington et al. 2022; Wang et al. 2025). Clinical metagenomics similarly stresses multidisciplinary review and clear separation of likely pathogens from incidental or contaminant signals (Chiu and Miller 2019; Wilson et al. 2019).

Comparative performance studies illustrate why this approach is necessary: detection in culture-negative samples and organism lists requiring exclusion decisions are common, and reporting must make these interpretive judgments explicit (Sun et al. 2025).

**Recommendation (Tier-1):** Reports should label findings as (a) confirmed, (b) presumptive pending confirmation, or (c) detected but not interpretable/actionable. Discordance between mNGS results and orthogonal methods (e.g., including scenarios where a targeted assay such as qPCR returns a positive result not supported by metagenomic signal) should be treated as an investigative trigger rather than resolved by automatic hierarchy. Neither platform's result is presumptively authoritative; discordance may reflect genuine differences in sensitivity, sample heterogeneity, inhibition, or methodological artifact, and the appropriate response is structured follow-up investigation with documentation of the discordant findings and their resolution.

Reports should include the evidentiary basis (controls, coverage metrics, confidence qualifiers), explicit analytical limitations (database scope, limits of detection, matrix effects), and recommended follow-up actions.

**SUPPLEMENTARY T2**

**Tier-2 applied metagenomics: the standard of evidence**

Tier-2 guidance applies to metagenomic applications intended to support population-level biosurveillance, situational awareness, and prioritization, including wastewater surveillance, environmental antimicrobial resistance (AMR) monitoring, pathogen-agnostic outbreak detection, and Scenario #3 early-warning use cases. Tier-2 outputs are designed to inform trend detection, risk prioritization, and hypothesis generation, rather than individual-level adjudication or regulatory enforcement. Accordingly, Tier-2 recommendations emphasize scalability, reproducibility, and longitudinal consistency, while explicitly bounding the interpretive claims that can be supported.

**Sample preparation and extraction**

Tier-2 programs should adopt matrix-appropriate, standardized sample preparation and extraction workflows tailored to the dominant sources of variability in the target environment (e.g., inhibitors, dilution effects, biomass heterogeneity). Absolute recovery efficiency is less critical than stable, reproducible processing conditions that support comparability across sites and timepoints. Where concentration or enrichment steps are employed, associated biases should be characterized and documented, and protocols should remain fixed within defined surveillance intervals to preserve longitudinal interpretability.

**Recommendation (Tier-2):**
Use standardized, representative-matrix-specific extraction workflows optimized for reproducibility rather than maximal sensitivity, and avoid mid-campaign protocol changes unless accompanied by overlap testing or recalibration.

**Controls and benchmarking**

Tier-2 control strategies are designed to support system-level normalization and background characterization, rather than exhaustive contamination adjudication. Negative controls, process blanks, and internal standards should be incorporated to quantify background signal, batch effects, and run-to-run variability. Unlike Tier-1, the presence of taxa in controls does not automatically preclude interpretation, but should inform background subtraction, signal qualification, and uncertainty estimation.

**Recommendation (Tier-2):**
Employ controls to characterize and normalize background signal at the system level and interpret detections in relation to comparative baselines rather than fixed suppression rules.

**Reference database selection**

Tier-2 surveillance programs face a distinct database governance challenge: because interpretation depends on longitudinal comparison rather than per-sample adjudication, database version stability is a prerequisite for analytical integrity. Apparent biological trends can be introduced or obscured by changes in database annotation, taxonomic reclassification, or contamination removal between builds (Jacobs 2026). Accordingly, Tier-2 database governance emphasizes consistency within surveillance windows over comprehensiveness at any single timepoint. Reference databases should be fixed for the duration of defined analytical periods, with version and provenance documented such that any future comparison can account for the database state at each timepoint. Database updates, reclassifications, or changes to contamination-screening procedures should be treated as potential confounders in longitudinal analysis and documented as such, with overlap analyses performed when transitions between database versions are necessary.

**Recommendation (Tier-2):**
Tier-2 database documentation requirements: 1) database name, build version, and access date; 2) scope and coverage relative to target taxa or functional gene families; 3) known annotation or contamination limitations affecting trend interpretation; and 4) a record of any version transitions within the surveillance period and the overlap analyses used to characterize their effect.

**Bioinformatics pipeline governance**

Tier-2 bioinformatics pipelines should be highly standardized and reproducible, emphasizing automation, containerization, and fixed parameters. Flexibility and per-sample optimization are deprioritized in favor of consistency across large datasets and multiple sites. Analytical outputs should focus on normalized counts, relative abundance, functional profiles, and aggregated metrics.

**Recommendation (Tier-2):**
Use validated, containerized pipelines with fixed parameters, and avoid analyst-driven tuning that compromises cross-study or longitudinal comparability.

**Signal validation and interpretation**

Validation in Tier-2 contexts is inherently comparative and contextual. Signals should be evaluated based on enrichment above background, consistency across replicates, temporal coherence, and concordance with external indicators such as clinical case trends or environmental drivers. Binary presence/absence thresholds are generally inappropriate.

**Recommendation (Tier-2):**
Interpret metagenomic signals as indicators of system-level change rather than definitive detections, and prioritize trend consistency and contextual alignment over per-sample confidence.

**Reporting and communication**

Tier-2 reporting should emphasize population-level trends, temporal dynamics, and spatial patterns, using graded confidence and transparent uncertainty communication. Results should be contextualized within broader surveillance frameworks and explicitly state analytical limitations.

**Recommendation (Tier-2):**
Report findings as trend-based indicators with clearly articulated uncertainty, and position metagenomic outputs as complementary inputs to broader surveillance and decision-support systems.

**SUPPLEMENTARY T3**

**Tier-3 applied metagenomics: the standard of evidence**

Tier-3 guidance applies to metagenomic applications oriented toward hypothesis generation, method development, novel pathogen discovery, and ecological characterization, where outputs are not expected to directly inform clinical, regulatory, or operational decisions. In contrast to Tier-1 and Tier-2 contexts, Tier-3 metagenomics is not governed by prescriptive control requirements or validation expectations; instead, the unifying requirement across all domains is disclosure. The objective is to ensure that methods, parameters, and interpretive context are recorded with sufficient fidelity to support reproducibility, reanalysis, and escalation to a higher tier if findings warrant further investigation. Tier-3 documentation requirements are therefore set not only by the immediate analytical context, but by the possibility that findings may later require higher-confidence adjudication.

**Sample preparation and extraction**

Tier-3 workflows permit broad flexibility in sample preparation, platform selection, and analytical approach. Ad hoc or novel protocols are acceptable, and platform choice is appropriately driven by cost, availability, and discovery objectives rather than regulatory defensibility. The documentation requirement is that workflows be described with sufficient detail for interpretability and reproducibility (e.g., what was done, with what materials and instruments, and under what conditions) such that another investigator could assess whether an observed signal might reflect a preparation artifact rather than a true biological finding.

**Recommendation (Tier-3):** Describe sample preparation and extraction methods with sufficient detail for reinterpretation, including extraction target, any enrichment or depletion steps applied, and known limitations of the chosen approach for the sample type.

**Controls and contamination**

Negative controls remain important at Tier-3, as contamination does not discriminate by application tier and can dominate signal in low-biomass and novel sample types. However, control results are used to contextualize findings rather than to enforce suppression rules. The presence of a taxon in a negative control should be disclosed and used to qualify reported detections; it does not automatically preclude interpretation but must be acknowledged as a potential source of artifact.

**Recommendation (Tier-3):** Include negative controls where feasible; document control results and acknowledge any taxa detected in controls that overlap with reported findings. Contamination risks should be discussed explicitly as part of result interpretation.

**Bioinformatics pipelines**

Tier-3 pipelines may be iteratively optimized as part of method development and are not required to be version-locked or containerized to the degree required at higher tiers. The documentation requirement is transparency: software names and versions, key parameter settings, reference resources used, and any branching decisions or manual review steps should be recorded with sufficient detail that the analytical logic can be reconstructed. This documentation supports both reanalysis and escalation. If a Tier-3 finding is later investigated under Tier-1 or Tier-2 standards, the original analytical environment must be recoverable enough to contextualize the initial detection.

**Recommendation (Tier-3):** Document software names and versions, key parameters, and any manual or analyst-driven steps. Methods should be described sufficiently for transparency even where formal reproducibility infrastructure such as containerization is not employed.

**Reference database governance**

Tier-3 database selection appropriately prioritizes breadth and inclusivity over the governance rigor required in higher-consequence contexts; broad, inclusive reference sets maximize discovery potential and hypothesis generation. However, Tier-3 database provenance must still be documented with sufficient fidelity to support later escalation. If a Tier-3 finding warrants investigation under Tier-1 or Tier-2 standards, the database state and version used in the original analysis must be recoverable, both to contextualize the initial detection and to identify whether a finding may reflect database artifact rather than true biological signal. A detection that cannot be traced to a specific, recoverable database version cannot be systematically reinterpreted as knowledge and reference resources evolve (Wright et al. 2024; Jacobs 2026).

**Recommendation (Tier-3):** Document database name and version or access date; characterize known scope limitations relevant to the organisms or signals detected; and include a statement of any known contamination or annotation concerns in the database relevant to reported findings.

**Signal validation and interpretation**

Tier-3 signal validation does not require orthogonal confirmation or formal adjudication. The standard of evidence is plausibility and novelty rather than definitive identification, and results should be framed as hypothesis-generating with clear statements of analytical limitations including database scope, sequencing depth, and known biases. Where a finding is sufficiently novel or concerning to warrant escalation (e.g., detection of a potential select agent or an unexpected engineered feature) it should be flagged for confirmation under Tier-1 or Tier-2 standards before informing operational decisions.

**Recommendation (Tier-3):** Frame findings explicitly as exploratory and hypothesis-generating. Report uncertainty and analytical limitations prominently. Define criteria for escalation to higher-tier review when findings meet a threshold of concern, and ensure documentation across all domains is sufficient to support that escalation pathway.

**SUPPLEMENTARY REFERENCES**

Alcock, B.P., Huynh, W., Chalil, R., Smith, K.W., Raphenya, A.R., Wlodarski, M.A., Edalatmand, A., Petkau, A., Syed, S.A., Tsang, K.K. and Baker, S.J., 2023. CARD 2023: expanded curation, support for machine learning, and resistome prediction at the Comprehensive Antibiotic Resistance Database. Nucleic Acids Research, 51(D1), pp.D690–D699.

Benoit, P., Brazer, N., de Lorenzi-Tognon, M., Kelly, E., Servellita, V., Oseguera, M., Nguyen, J., Tang, J., Omura, C., Streithorst, J. and Hillberg, M., 2024. Seven-year performance of a clinical metagenomic next-generation sequencing test for diagnosis of central nervous system infections. Nature Medicine, 30(12), pp.3522–3533.

Chan, A.P., Siddique, A., Desplat, Y., Choi, Y., Ranganathan, S., Choudhary, K.S., Diaz, J., Bezney, J., DeAscanis, D., George, Z. and Wong, S., 2022. A universal day zero infectious disease testing strategy leveraging CRISPR-based sample depletion and metagenomic sequencing. medRxiv, pp.2022–05.

Child, H.T., Airey, G., Maloney, D.M., Parker, A., Wild, J., McGinley, S., Evens, N., Porter, J., Templeton, K., Paterson, S. and Van Aerle, R., 2023. Comparison of metagenomic and targeted methods for sequencing human pathogenic viruses from wastewater. mBio, 14(6), e01468-23.

Dong, W., Fan, X., Guo, Y., Wang, S., Jia, S., Lv, N., Yuan, T., Pan, Y., Xue, Y., Chen, X. and Xiong, Q., 2024. An expanded database and analytical toolkit for identifying bacterial virulence factors and their associations with chronic diseases. Nature Communications, 15(1), p.8084.

Grimm, S.L., Rothman, J.A., Bradshaw, W.J., Langlois, K., Steele, J.A., Griffith, J.F., Kaufman, J.T. and Whiteson, K.L., 2025. Deep metatranscriptomic sequencing data of wastewater from Los Angeles, USA, 2023–2024. Scientific Data.

Harmon, C.L., Castlebury, L.A., Boundy-Mills, K., Broders, K.D., Hyten, A.M., Jacobs, J.L., Knight-Connoni, V.K., Mollov, D., Riojas, M.A. and Sharma, P., 2023. Standards of diagnostic validation: recommendations for reference collections. PhytoFrontiers, 3(1), pp.43–50.

Hopkins, L., Ensor, K.B., Stadler, L., Johnson, C.D., Schneider, R., Domakonda, K., McCarthy, J.J., Septimus, E.J., Persse, D. and Williams, S.L., 2023. Public health interventions guided by Houston’s wastewater surveillance program during the COVID-19 pandemic. Public Health Reports, 138(6), pp.856–861.

Konganti, K., Reed, E., Mammel, M., Kayikcioglu, T., Binet, R., Jarvis, K., Ferreira, C.M., Bell, R.L., Zheng, J., Windsor, A.M. and Ottesen, A., 2023. bettercallsal: better calling of Salmonella serotypes from enrichment cultures using shotgun metagenomic profiling and its application in an outbreak setting. Frontiers in Microbiology, 14, p.1200983.

Nguyen, S.V., Puthuveetil, N.P., Petrone, J.R., Kirkland, J.L., Gaffney, K., Tabron, C.L., Wax, N., Duncan, J., King, S., Marlow, R., Reese, A.L., Yarmosh, D.A., McConnell, H.H., Fernandes, A.S., Bagnoli, J., Benton, B. and Jacobs, J.L., 2024. The ATCC genome portal: 3,938 authenticated microbial reference genomes. Microbiology Resource Announcements, 13(2), e01045-23.

Philo, S.E., De León, K.B., Noble, R.T., Zhou, N.A., Alghafri, R., Bar-Or, I., Darling, A., D’Souza, N., Hachimi, O., Kaya, D. and Kim, S., 2024. Wastewater surveillance for bacterial targets: current challenges and future goals. Applied and Environmental Microbiology, 90(1), e01428-23.

Ritter, V., Kircher, S., Sturm, K., Warns, P. and Dick, N., 2009. USDA FSIS, FDA BAM, AOAC, and ISO culture methods BD BBL CHROMagar Listeria Media. Journal of AOAC International, 92(4), pp.1105–1117.

Wang, Q., Kille, B., Liu, T.R., Elworth, R.L. and Treangen, T.J., 2021. PlasmidHawk improves lab of origin prediction of engineered plasmids using sequence alignment. Nature Communications, 12(1), p.1167.

Wang, J., den Bakker, H.C. and Denes, T.G., 2025. A critical review of metagenomic approaches for foodborne pathogen surveillance. Critical Reviews in Food Science and Nutrition, 65(33), pp.8488–8501.

Wilson, M.R., Naccache, S.N., Samayoa, E., Biagtan, M., Bashir, H., Yu, G., Salamat, S.M., Somasekar, S., Federman, S., Miller, S. and Sokolic, R., 2014. Actionable diagnosis of neuroleptospirosis by next-generation sequencing. New England Journal of Medicine, 370(25), pp.2408–2417.

Wilson, M.R., Sample, H.A., Zorn, K.C., Arevalo, S., Yu, G., Neuhaus, J., Federman, S., Stryke, D., Briggs, B., Langelier, C. and Berger, A., 2019. Clinical metagenomic sequencing for diagnosis of meningitis and encephalitis. New England Journal of Medicine, 380(24), pp.2327–2340.

Wittmann, B.J., Alexanian, T., Bartling, C., Beal, J., Clore, A., Diggans, J., Flyangolts, K., Gemler, B.T., Mitchell, T., Murphy, S.T. and Wheeler, N.E., 2025. Strengthening nucleic acid biosecurity screening against generative protein design tools. Science, 390(6768), pp.82–87.

Zhou, S., Liu, B., Zheng, D., Chen, L. and Yang, J., 2025. VFDB 2025: an integrated resource for exploring anti-virulence compounds. Nucleic Acids Research, 53(D1), pp.D871–D877.

Zolfo, M., Tett, A., Jousson, O., Donati, C. and Segata, N., 2017. MetaMLST: multi-locus strain-level bacterial typing from metagenomic samples. Nucleic Acids Research, 45(2), e7.
